# The Utility of *Capsicum annuum* L. in Internal Medicine and In Dentistry: A Comprehensive Review

**DOI:** 10.3390/ijerph191811187

**Published:** 2022-09-06

**Authors:** Luciano Maria Catalfamo, Giulia Marrone, Michele Basilicata, Ilaria Vivarini, Vincenza Paolino, David Della-Morte, Francesco Saverio De Ponte, Francesca Di Daniele, Domenico Quattrone, Danilo De Rinaldis, Patrizio Bollero, Nicola Di Daniele, Annalisa Noce

**Affiliations:** 1 Department of Biomedical and Dental Sciences, Morphological and Functional Images, University Hospital of Messina, 98100 Messina, Italy; 2 UOC of Internal Medicine-Center of Hypertension and Nephrology Unit, Department of Systems Medicine, University of Rome Tor Vergata, 00133 Rome, Italy; 3 UOSD Special Care Dentistry, Department of Experimental Medicine and Surgery, University of Rome Tor Vergata, 00100 Rome, Italy; 4 UOSD Special Care Dentistry, Department of Systems Medicine, University of Rome Tor Vergata, 00100 Rome, Italy; 5 Department of Systems Medicine, University of Rome “Tor Vergata”, 00133 Rome, Italy; 6 Department of Human Sciences and Quality of Life Promotion, San Raffaele University, 00166 Rome, Italy; 7 Department of Neurology, Evelyn F. McKnight Brain Institute, Miller School of Medicine, University of Miami, Miami, FL 33136, USA; 8 School of Applied Medical, Surgical Sciences, University of Rome Tor Vergata, 00133 Rome, Italy; 9 UOSD of Dermatology, Department of Systems Medicine, University of Rome Tor Vergata, 00133 Rome, Italy

**Keywords:** *Capsicum annuum* L., Capsaicin, internal medicine, nephropathy, dentistry, oro-facial pain, topical application, neuropathic oral diseases

## Abstract

Capsaicin is a chili peppers extract, genus Capsicum, commonly used as a food spice. Since ancient times, Capsaicin has been used as a “homeopathic remedy” for treating a wild range of pathological conditions but without any scientific knowledge about its action. Several studies have demonstrated its potentiality in cardiovascular, nephrological, nutritional, and other medical fields. Capsaicin exerts its actions thanks to the bond with transient receptor potential vanilloid subtype 1 (TRPV1). TRPV1 is a nociceptive receptor, and its activation starts with a neurosensitive impulse, responsible for a burning pain sensation. However, constant local application of Capsaicin desensitized neuronal cells and leads to relief from neuropathic pain. In this review, we analyze the potential adjuvant role of Capsaicin in the treatment of different pathological conditions either in internal medicine or dentistry. Moreover, we present our experience in five patients affected by oro-facial pain consequent to post-traumatic trigeminal neuropathy, not responsive to any remedy, and successfully treated with topical application of Capsaicin. The topical application of Capsaicin is safe, effective, and quite tolerated by patients. For these reasons, in addition to the already-proven beneficial actions in the internal field, it represents a promising method for the treatment of neuropathic oral diseases.

## 1. Introduction

**Figure 1:**
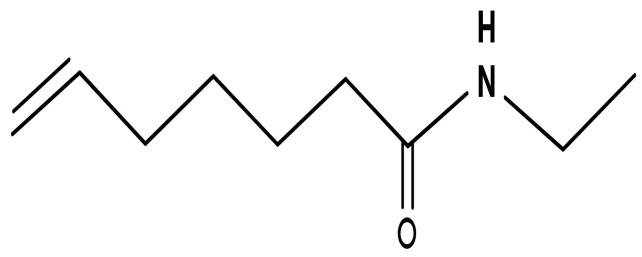
Capsaicin chemical structure.

Capsaicin is an active ingredient found in common spices such as chili peppers and similar plants, belonging to the *Capsicum annuum* L. family (Figure 1).

Chili pepper contains between 0.1 and 2.0% of Capsaicin [1]. Capsaicin is the most powerful element of the Capsicum family and has painful, pungent, and desensitizing effects, due to the presence in the molecule of an amidic bond and a double bond [2]. Capsaicin was firstly isolated in 1816 by Christian Bucholz [3], a German pharmaceutical chemist. Since ancient times, Capsaicin has been used as a homeopathic remedy to treat a wide range of pathological conditions, according to the concept of “treating like with like” [3] or “fight fire with fire” [1].

**Figure 2:**
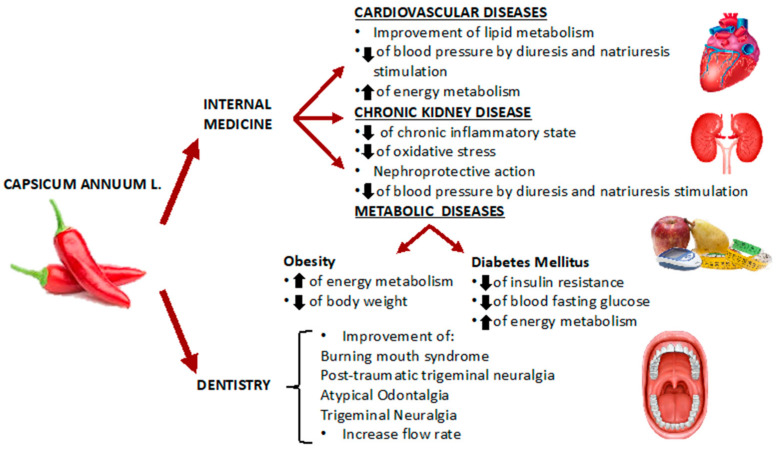
*Capsicum annuum* L. and its potential use in internal medicine and in dentistry.

Several studies have been conducted in order to assess the efficacy of Capsaicin in the treatment of different pathologies (Figure 2).

A study conducted by Yeam et al. [4] highlighted that topical use of Capsaicin is one of the most used alternative medicine remedies for the treatment of uremic pruritus in chronic kidney disease (CKD) patients.

Blair et al. [5] demonstrated that a single 60-min application of an 8% Capsaicin dermal patch provided rapid and substantial pain relief in patients with post-herpetic neuralgia.

Campbell et al. [6] investigated Capsaicin’s local infiltration and showed that it represents a promising medical treatment for knee osteoarthritis pain.

Bryniarska et al. [7] demonstrated that 8% Capsaicin patches are effective for the treatment of pain associated with chemotherapy-induced neuropathy in oncological patients in treatment with oxaliplatin.

Watson et al. [8] observed, in a randomized clinical trial, the efficacy of topical application of 0.075% Capsaicin cream in women with post-mastectomy pain syndrome.

Berger et al. [9] demonstrated that oral Capsaicin, in a taffy candy vehicle, induced significant temporary relief of pain in patients with oral mucositis secondary to chemotherapy and radiation therapy.

Romero et al. [10] reported that the local application of 8% Capsaicin cream is beneficial and well-tolerated by patients with myofascial pain syndrome.

Kim et al. [11] demonstrated that intravesical instillation of Capsaicin, or Resiniferatoxin (an ultra-potent analogue of Capsaicin), is a promising treatment for the overactive bladder.

Gálvez et al. [12] showed that an 8% Capsaicin patch is a well-tolerated and long-term effective adjuvant in the treatment of post-herpetic neuropathy (PHN), post-traumatic or post-surgical nerve injury, and HIV-associated distal sensory polyneuropathy (HIV-DSPN).

Zebda et al. [13] described that 0.1 mM intranasal Capsaicin spray improves rhinitis symptoms and also reduces nasal reactivity and nasal congestion after a 12-week treatment.

Jankovskis et al. [14] reported that oral rinses with 0.02% Capsaicin, associated with vitamin B and zinc oral supplements, can be helpful in patients affected by burning mouth syndrome (BMS) [15,16].

## 2. Material and Methods

We conducted a literature search looking at the relationship between keywords such as “capsaicin” [Title/Abstract] in combination with “internal medicine” or “dentistry” and “natural bioactive compounds” and/or “diabetes mellitus” and/or “chronic kidney disease” and/or “metabolic syndrome” and/or “arterial hypertension” and/or “oral health” and/or “oral cancer” and/or “burning mouth syndrome” and/or “periodontal health” on PubMed, Scopus, and Web of Science electronic databases.

The reference lists and the related records were manually reviewed by the authors. The search was limited to English language papers published up to July 2022.

## 3. Mechanism of Action

Capsaicin is a member of chemical compounds belonging to the vanilloid family (Figure 1). Capsaicin acts as an agonist to transient receptor potential vanilloid (TRPV) 1, which is a non-selective cation channel, widely expressed in sensory nerve fibers, with a high affinity for calcium. TRPV1 is present in several non-neural cells, including vascular endothelial, smooth muscle, and renal cells [17]. Overall, the TRPV subfamily consists of six members and is divided into four subunits, according to their homology: TRPV1/TRPV2, TRPV3, TRPV4, and TRPV5/TRPV6 [17]. All of these protein receptors share the same molecular structure, composed of six regions of the transmembrane, forming a non-specific ionic channel [14]. TRPV1 is the first member of the TRPV subfamily that was discovered by Caterina et al. in 1997 [15]. The authors cloned a receptor that can be activated by Capsaicin, thus, TRPV1 is also known as “Capsaicin receptor” [18].

TRPV1 is a polypeptide transmembrane receptor composed of 838 amino acids, with relatively modest Ca^2+^ permeability. It presents a tetrameric structure consisting of six transmembrane regions (TM1-6) with a hydrophobic group, located between the fifth and sixth transmembrane regions (TM5/6) [19]. Both the N-terminal and the C-terminal are located on the inner side of the cell membrane and regulate the functional activities of the receptor [19].

N-terminal region, collected by 432 amino acids, contains at least three ankyrin repeats, which are essential for the channel function. The C-terminal region, composed of 154 amino acids, presents a TRP domain, consisting of 25 amino acids, likely with the function of determining subunits tetramerization [19]. Both C- and N-terminal regions and the intracellular loop, connecting TM2 and TM3, contain PKA/PKC (protein kinase A and C) and phosphorylation sites. The C-terminal region contains the phosphatidylinositol 4,5-biphosphate binding domain. Both C-terminal and N-terminal regions contain calmodulin-binding sites [19].

**Figure 3:**
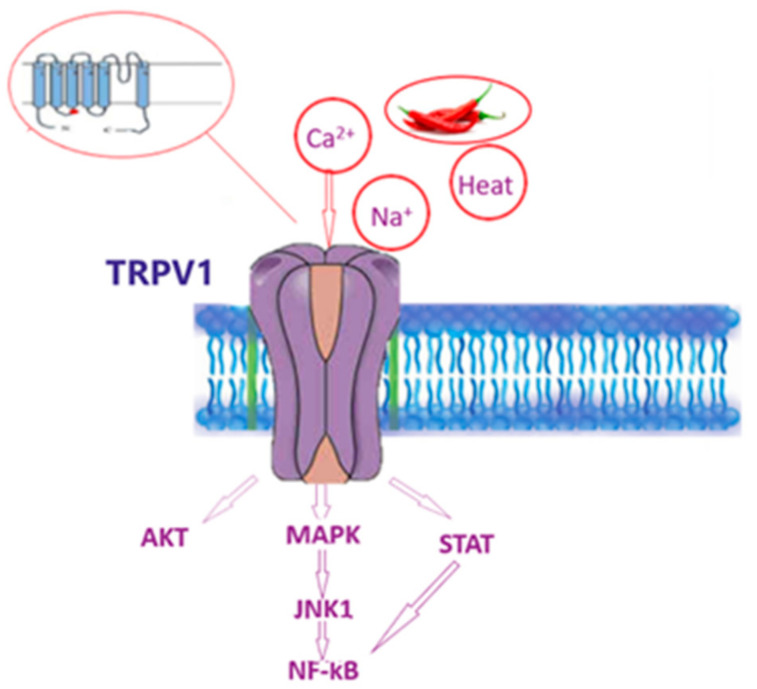
Representation of TRPV1 receptor and the main mechanisms of action. TRPV1 acts on three pathways: Phosphoinositide 3-kinase/protein kinase B (PI3/AKT), Transforming growth factor-β-activated kinase 1/mitogen-activated protein-kinase (TAK1/MAPK), and Janus kinase/signal transducer and activator of transcription (JAK/STAT). In particular, the calcium influx stimulates TRPV1 and improves the activation of several kinases that promote the activation of nuclear factor-kB (NF-kB).

The structure of TRPV1 was analyzed by Moiseenkova-Bell et al. [20], using cryo-electron microscopy. The authors found that the Capsaicin receptor/channel is composed of two distinct regions: a large open basket-like domain, corresponding to N- and C-terminal cytoplasmatic portions, and a compact domain, corresponding to the transmembrane portion. The arrangement of these two domains forms a structure named “hanging gondola”, in which the cytoplasmic region “hangs” underneath the transmembrane domain of the channel [17,20]. Capsaicin interacts with the cytoplasmatic domain of the third and the fourth transmembrane proteins of TRPV1 [17]. The activation of this receptor induces Calcium influx, which in turn triggers the three major pathways, namely phosphoinositide 3-kinase/protein kinase B (PI3/AKT), transforming growth factor-β-activated kinase 1/mitogen-activated protein-kinase (TAK1/MAPK) and Janus kinase/signal transducer and activator of transcription (JAK/STAT) (Figure 3).

TRPV1 ion influx leads to the activation of nuclear factor-kB (NF-kB), until the transcription of nucleus target genes [21]. The activation of these pathways increases the neuronal cells’ sensibility, via the synthesis of new TRPV1 receptors and their translocation on the cell membrane [21]. Several agents can activate the TRPV1 receptor’s physical factors, such as a high temperature (over 42 °C), and chemical factors, such as acid solution (pH < 6.5) and endogenous anti-inflammatory lipids (like prostaglandins and leukotrienes) [3].

When Capsaicin (or other agents) activates the TRPV1 receptor, cellular ion calcium influx occurs. This phenomenon induces a depolarization along the fibers, thus propagating the burning signal into the spinal cord up to the brain, thanks to the neurotransmitter substance P (SP) [3].

Even if Capsaicin activates TRPV1, its continuative use desensitizes the sensory afferent axons, reducing pain transmission. Currently, there are no scientific explanations for this phenomenon. In this regard, three options have been proposed:

- Repeated applications of Capsaicin deplete neurons’ SP [22].
- An excess of ion calcium influx causes the loss of mitochondrial functions, making the afferent fiber inoperative [23].
- High levels of intracellular calcium have cytotoxic effects, leading to the selective death of neuronal cells expressing TRPV1 receptors [24].

Likely, more than one of the mechanisms is responsible for the analgesic effect of Capsaicin’s long-term local application. However, the analgesic effect of a continuative application of Capsaicin is totally reversible several times after its treatment withdrawal [25].

The reason behind the reversible analgesic effect of Capsaicin could be that it does not cause the death of neurosensitive fibers, but just the terminal dendritic degeneration of neuronal cells. After its suspension, these thin distal branches, thanks to their great re-growing capability, resume their initial position and recover their full functionality.

## 4. Pharmacological Aspects

Capsaicin can be administrated in several forms: As a gel, cream, ointment, patch, spray, infiltration solution, mouthwash, and candy [3].

The pharmacological aspects of Capsaicin are very interesting. In fact, it is true that Capsaicin is able to produce a burning sensation up to pain, and if it is administered at a high or repeated dose, it can induce analgesia [26]. This action is exerted through the loss of sensitivity to “painful stimuli”.

Currently, the exact mechanisms at the base of Capsaicin-induced desensitization are not well-known. Several authors have shown the depletion of SP, a neuropeptide involved in the nociceptor signal of the fibers that express TRPV1 and increased ion calcium intracellular levels, causing the inhibition of calcium channels [27,28]. As a result of the calcium intracellular accumulation, TRPV1 is inhibited by the activation of calcium-dependent proteins and desensitization appears [29].

Another proposed Capsaicin mechanism of action concerns the inhibition of Piezo proteins by TRPV1, which is related to the depletion of phosphoinositides [30]. The decrease in phosphoinositides is correlated, in turn, with the inhibition of Piezo channels and thus the inhibition of inward current. The latter mechanism is able to explain, in part, Capsaicin’s mechanical analgesia [30].

There are no absolute contraindications to the use of Capsaicin, but its treatment has had to be suspended in case of poor tolerance by patients. The main side effects related to Capsaicin’s local application are erythema, burning, swelling, pruritus, and hyperesthesia [3]. Another contraindication consists of bronchial asthma, as these patients show greater sensitivity to Capsaicin [3]. Finally, patients must be careful to avoid ocular contact, as it can cause burning pain, tearing, photophobia, and blurry vision [3].

The first approval, in 2009, by the Food and Drug Administration (FDA) concerned an 8% Capsaicin patch (Qutenza) for the treatment of neuropathic pain associated with post-herpetic neuralgia. In 2020, the FDA approved Capsaicin for the treatment of neuropathic pain associated with peripheral neuropathy that causes “diabetic foot” [31].

## 5. Effects of *Capsicum annuum* L. in Internal Medicine

Capsaicin, the main active substance of red pepper, has been shown to have therapeutic applications. In fact, as discussed above, topically, it can exert a pain-relieving effect in arthritis, post-operative neuralgia, diabetic neuropathy, psoriasis, etc. Moreover, the systemic administration of Capsaicin can induce cardioprotective effects, anti-lithogenic action, anti-inflammatory, analgesic, and thermogenic effects, and exert a beneficial impact on the gastrointestinal system [32]. Chinense and Frutescens species contain many antioxidant metabolites, but the highest amount of antioxidants is contained in *Capsicum annuum* L. [2]. The main actions of Capsaicin are anti-pain, anti-inflammatory, and antioxidant. Therefore, thanks to its properties, several studies investigate the usefulness of Capsaicin in the treatment of chronic inflammatory diseases [33]. In fact, the fruit of this family contains many capsaicinoids, carotenoids, flavonoids, vitamins, and minerals that are able to counteract the inflammatory processes. The type and quantity of these compounds vary according to the cultivar, the fruit ripening, or the cultivation system [2]. Therefore, these metabolites could be considered a natural defense against various illnesses.

Studies in vitro showed that Capsaicin can inhibit interleukin-6, E2 prostaglandins, and tumor necrosis factor-alpha (TNF-α). Other evidence highlights the role of *Capsicum baccatum* L. juice in counteracting cytokine production in the inflammatory site, through a reduction in neutrophils and cytokines recruitment in the exudate [34].

Recent studies confirmed that chili consumption is inversely related to mortality, obesity [35], arterial hypertension [36], and CKD [32,33]. Regarding the latter, some studies in vitro on HK-2 cells and in vivo in animal models showed that Capsaicin increased heme oxygenase-1 (HO-1) expression, the enzyme involved in heme metabolism [37]. In fact, this enzyme can exert a nephroprotective action against cisplatin, oxidative stress, and inflammation. Animal studies demonstrated that Capsaicin could act as a diuretic agent, modulating the expression of epidermal growth factor (EGF) in diabetic mice models compared to healthy mice. Furthermore, the activation of the Capsaicin receptor TRPV1 induces the improvement of the glomerular filtration rate (GFR), the prevention of salt-induced kidney damage, and the enhancement of natriuresis and diuresis [38,39,40]. Another study conducted on an animal model by Jung et al. reported that Capsaicin could reduce renal damage, inflammation, and oxidative stress [37].

Currently, there are few human studies that demonstrate the beneficial role of Capsaicin in CKD. Rare human studies are available, and they investigate its beneficial effects on obesity and arterial hypertension, which represent risk factors for CKD onset. In fact, only one study demonstrated that chili intake was inversely associated with CKD onset, among participants of the China Health and Nutrition Survey (CHNS). This association was independent of lifestyle, other comorbidities, gender, or demographic backgrounds [33].

A recent study by Noce et al., conducted on CKD male patients, demonstrated that an oral food supplement, based on natural bioactive compounds, among these *Capsicum annuum* L., is a possible adjuvant treatment in the management of CKD-related comorbidities [34,41]. In particular, after 5 weeks of 3 cps daily assumption of this oral food supplement, the authors observed an amelioration in the lipid profile, inflammatory status, and systolic and diastolic blood pressure [42]. Moreover, the data showed an enhancement in total-body water and extracellular and intracellular water distribution.

Cardiovascular diseases (CVDs) represent the leading cause of death in the world. Moreover, the presence of comorbidity increases their progression and mortality risk [43]. One of its comorbidities is dyslipidemia. Different studies have shown that Capsaicin is able to enhance high-density lipoproteins (HDL) and reduce triglycerides (TG), low-density lipoproteins (LDL), and total cholesterol [32]. In light of these facts, the assumption is that red pepper induces a reduction in lipidic absorption in the gut, an increase in cholesterol elimination through the intestinal route, and the activation of peroxisome proliferators-activated receptor alpha (PPAR-α), a factor that increases HDL and reduces TG [44,45]. The first studies that pointed out the antilipemic action of Capsaicin were performed in vitro. In particular, they demonstrated that chili pepper could transactivate PPAR-α, and thanks to its antioxidant properties, could counter LDL oxidation [46]. Animal studies confirmed previous data. In fact, in mice with an 8-week high-fat diet, Capsaicin significantly reduced the total level of TG [47]. Another study showed that the assumption of 200 mg/kg of red pepper aqueous extract decreased body weight, total cholesterol, TG, and LDL and increased HDL [48]. Clinical in vivo and in vitro studies reached the same conclusions, so Capsaicin represents an adjuvant treatment for dyslipidemia and, in turn, reduces cardiovascular risk [49,50].

Another cardiovascular risk factor is arterial hypertension (AH), and Capsaicin can interfere in its etiopathogenesis, releasing vasodilator neuropeptides through TRPV1 activation, inhibiting L-type Ca^2+^ channels in smooth muscle cells, stimulating natriuresis and diuresis, and inhibiting the angiotensin-converting-enzyme (ACE). In fact, in in vitro studies, red pepper was used as an inhibitor of ACE. Moreover, in animal models, it was observed that treatment with 5 mg/mL of Capsaicin in hypertensive mice improved the mean systemic arterial blood pressure [36]. Another in vivo study revealed that the anti-hypertensive and anti-tachycardic effects of Capsaicin are the result of sensitive neuron stimulation, which induces the release of hypotensive peptides [51,52].

Capsaicin, through TRPV1, exerts an endothelial protective action, inducing the release of nitric oxide (NO) and calcitonin gene-related peptide (CGRP). TRPV1 is present in many metabolically active tissues, making them potential targets for the treatment of dysmetabolic diseases [53]. Comparing short- and long-term administration of Capsaicin in genetically hypertensive rats, the results showed that, in both cases, it is able to lower blood pressure. In acute administration, it causes the enhancement of CGRP plasma levels [54]. Regarding clinical studies, there is not enough evidence to confirm the results obtained in in vitro and in vivo studies.

Diabetes mellitus (DM) is another chronic degenerative non-communicable disease in which the efficacy of Capsaicin was observed [55]. Capsaicin seems to have insulin-mimetic activity. Moreover, it seems to exert an antioxidant action to regulate body weight and suppress inflammation. Thus, it could be considered a good glucose homeostasis regulator [56]. In vitro studies demonstrated that Capsaicin increased glucose uptake in C2C12 muscle cells thanks to the activation of the AMP-activated protein kinase (AMPK) [57]. Its interaction with intestinal cells inhibits glucose absorption, but this effect is dependent on the concentration and incubation time of Capsaicin [58]. Pre-treatment with Capsaicin seemed to increase energy metabolism in human intestinal and epithelial cells, thanks to the enhanced expression of the glycolytic enzymes triosephosphate isomerase and phosphoglycerate mutase [59]. Capsaicin increases the plasmatic insulin concentration and reduces the blood glucose concentration in dog animal models. In a study conducted on mice that followed a 10-week high-fat diet, Capsaicin treatment reduced fasting glucose, leptin, insulin, and inflammatory markers in the liver and adipose tissue. Therefore, Capsaicin seems to be a valid and promising adjuvant treatment for insulin resistance.

An improvement in hepatic insulin sensibility was also correlated with the increase in hepatic glycogen storage and the reduction in hepatic glucose output [60]. In streptozotocin-induced diabetic rats, Capsaicin activated TRPV1 in the pancreas and in liver cells, causing an increase in TRPV1, pancreatic duodenal homebox-1 (PDX-1), and liver X receptor (LXR) expression. LXR and PDX-1 are important factors in glucose homeostasis; in fact, they can regulate the expression of glucokinase, phosphoenolpyruvate carboxykinase, glucose transporter 2 (GLUT2), and glucose 6-phosphatase [61]. In diabetic and KK-A^y^ genetically obese mice, Capsaicin reduced blood glucose, triglyceride, and insulin concentrations, as well as hepatic triglyceride deposition. Its assumption seems to counteract inflammation in the adipose tissue, without body weight variations [62]. In a Zucker mice study, the desensitization of sensory nerves through Capsaicin improved oral glucose tolerance and fasting glucose concentrations. These results were obtained without any variation in blood insulin, glycogen, and triglyceride concentrations [63]. In a study on wild-type mice, Capsaicin increased GLP-1 and insulin secretion, both with acute and chronic administration [64].

Clinical trials confirmed the results obtained in animal and in vitro studies. In particular, research conducted on healthy subjects demonstrated that Capsaicin could improve gut glucose absorption and glucagon release during the glucose loading test [65]. In another study on healthy people, 5 g/day of Capsaicin decreased the glucose blood concentration and increased insulin blood levels [66]. A further study conducted on long-distance male runners showed that a single meal with 10 g of red pepper promoted carbohydrate catabolism thanks to the increase in plasma epinephrine [67].

A 4-week treatment of chilies in women with gestational diabetes caused a reduction in post-prandial hyperinsulinemia and hyperglycemia with an improvement in lipid metabolism [68]. So, Capsaicin has a relevant role in DM therapy, but other studies with larger populations are necessary to confirm its potential efficacy.

Obesity is defined as abnormal or extra-fat accumulation, characterized by an excess of adipose cells. Therefore, this pandemic condition is strictly correlated to DM, dyslipidemia, and CVDs onset, and obesity is a risk factor for metabolic syndrome [69].

Several studies have shown that Capsaicin was effective in body weight loss because it promotes thermogenesis and satiety, increases energy expenditure [35] and fat oxidation [70], prevents adipogenesis [32], reduces energy intake [71], activates pancreatic lipase [72] and lipoprotein lipase [69], modulates adipokine release [73], and stimulates lipolysis [74]. Regarding thermogenesis, Capsaicin could induce the browning effect in white adipose cells [75]. Its interaction with adipose tissues stimulates the expression of PPAR-α, PPAR-γ, UCP1, PRDM-16, and SIRT1, which are involved in thermogenesis [76].

In fact, in vitro studies confirmed these activities as observed in 3T3-L1 preadipocytes and adipocytes cell lines. As a matter of fact, a low dosage of Capsaicin reduced C/EBP-α, PPAR-γ, and leptin expression. In the meantime, it promoted anti-adipogenic genes and apoptosis and inhibited adipogenesis. Meanwhile, a high dosage of Capsaicin stimulated adipogenesis with a low expression of anti-adipogenic genes [77,78]. In Caco-2 cells, without the activation of TRPV1, Capsaicin reduced the absorption of fatty acids and increased the stimulation of acetyl-coenzyme A synthetase [79]. Other studies showed that *Capsicum annum* L. extracts could inhibit pancreatic lipase, which is responsible for TG hydrolyzation [72].

Mice that followed a diet containing 0.0014% Capsaicin without any caloric change obtained a reduction in body weight, particularly related to visceral fat loss [80]. Different research studies confirmed that Capsaicin can suppress obesity-induced inflammation by regulating adipokine production in obese mice [73]. Human studies confirmed previous results; in fact, a combination of green tea, Capsaicin, and ginger in overweight women caused a body weight loss, a reduction in body mass index and serum insulin, and an improvement in the homeostasis model assessment of insulin resistance (HOMA-IR) [81]. In Caucasian subjects, it has been observed that 2.56 mg of Capsaicin in every meal increased satiety [82].

Interestingly, it has been demonstrated that Capsaicin can generate body weight loss not only with oral administration but also through both cerebral injection and topical application [83]. Regarding topical application, Capsaicin is able to reduce body weight and inflammation, and it can increase insulin sensibility without any caloric restriction [84]. In another mice study, the desensitization of Capsaicin-sensitive nerves was able to prevent aging-induced obesity up to one year after treatment [85].

## 6. Effects of *Capsicum annuum* L. in Dentistry

**Figure 4:**
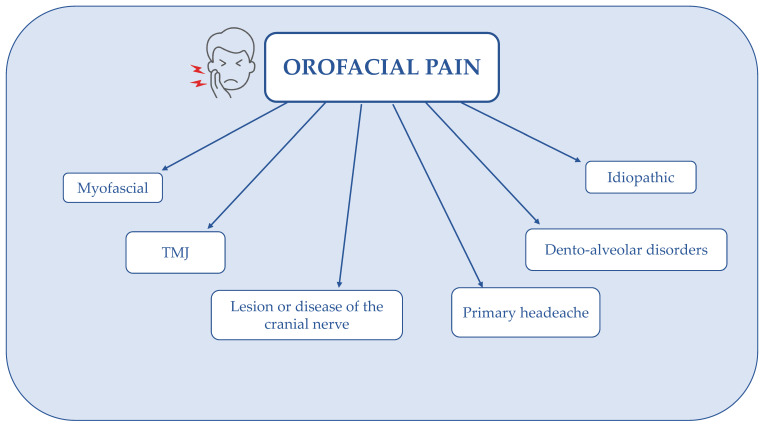
The etiology of pain in the oral mucosa according to the International Classification of Orofacial Pain (ICOP). Abbreviations: TMJ, temporomandibular joint.

The oral cavity is a dynamic environment continually subjected to mechanical, thermal, and chemical stimuli, due to food ingestion and chewing. The oral mucosa exhibits a sensory nature similar to the face skin and gut. In fact, like the face skin, the oral mucosa requires a high level of sensitivity to several stimuli related to the quali-quantitative composition of foods, useful to prevent the ingestion of harmful foods [86]. On the other hand, like the gut, the oral mucosa must be insensitive to the mechanical chewing of hard foods or hot drinks. In addition, multiple causes induce acute or chronic pathological pain in the oral cavity, which can critically affect the quality of life by impairing vital functions such as eating and swallowing (Figure 4) [87].

Capsaicin performs its analgesic effects thanks to the bond with TRPV1, which is present in the oral mucosa. In fact, it has been observed that an injection of Capsaicin might provide a localized numbness without the loss of muscle control caused by traditional anesthetics such as procaine. Treatment with Capsaicin is effective in different types of painful conditions such as complex regional pain syndromes and neuropathic pain. The combination of the local anesthetic lidocaine-derived QX-314 with Capsaicin, applied to a sensory nerve, produces long-lasting analgesia in the orofacial area and, in an animal model, seems to inhibit the jaw-opening reflex induced by the stimulation of the tooth pulp [88].

Regarding somatosensory transmission, the nociception in orofacial tissues is due to the trigeminal nerve (V cranial nerve). Trigeminal ganglia have the cell bodies of sensory neurons, which project both centrally and peripherally. Nociceptive nerves thus contract synapses with second-order neurons within the trigeminal nucleus complex, particularly in the caudal area [89]. This area transmits nervous signals to brain regions involved in sensory discrimination and somatic pain. Some microneurographic studies in humans have shown that mechanoreceptors in the oral mucosa have both fast and slow adaptive abilities [90]. Furthermore, in animal models, the oral mucosa has three types of mechanoreceptors: Meissner corpuscles, Ruffini endings, and Merkel cells, while Pacinian corpuscles are not present [90]. Nociceptive fibers are classified as Aδ (58%) or C (42%) fibers according to their transmission rate. In rodents, TRPV1 and TRPA1 are expressed by approximately 40–45% of the buccal mucosal afferent fibers [91]. These receptors can transduce the burning pain caused by Capsaicin and mustard oil, with CGRP release [92]. Besides, the oral mucosa is innervated by the gustatory nerves, namely the chorda tympani nerve and the glossopharyngeal nerve. It is known that taste modulates pain. For example, sucrose ingestion is effective in relieving intraoral pressure or cold pain in infants [93] and reducing the activation of certain brain regions in adults. Conversely, chronic pain conditions affect taste sensations, inducing, in patients with BMS, a decrease in taste compared to healthy subjects [94]. In addition, these patients exhibit hypofunction of the chorda tympani nerve.

**Figure 5:**
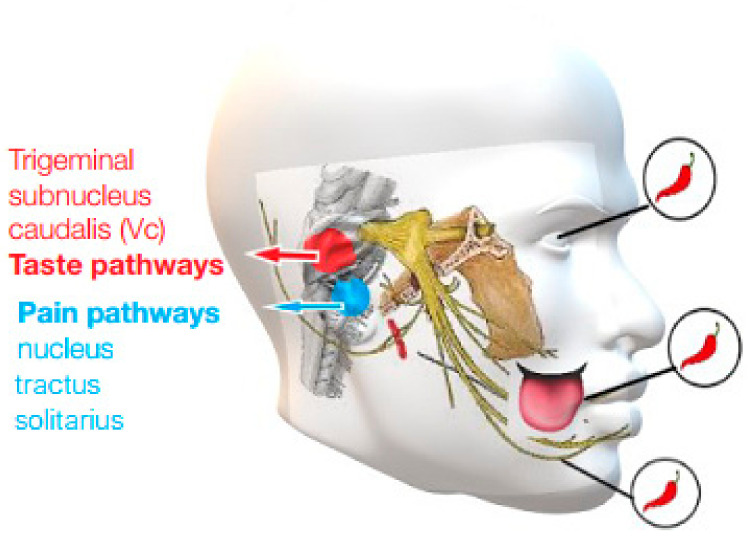
Sites of Capsaicin injection into ophthalmic, maxillary, and mandibular branches.

Neurons in the trigeminal caudal nucleus (Vc) are critical centers for oral and facial pain transmission [95] and they are activated by a multitude of stimuli. Therefore, the application of different substances has been evaluated, such as Capsaicin, ethanol, histamine, mustard oil, nicotine, acid, and piperine [89]. Specifically, the mapping of the extracellular signal-regulated phosphorylated kinase (pERK), following Capsaicin injection into various orofacial areas, revealed the somatotopic distribution of neurons in the Vc, upper cervical spinal cord, and intraoral and extraoral chemical transmission pathways [96] (Figure 5). Extraorally, Capsaicin injection into the ophthalmic, maxillary, or mandibular regions stimulates pERK+ neurons in the ventral, middle, and dorsal portions of the Vc. On the contrary, Capsaicin injection into the oral mucosa does not show pERK+ neuron localization. The injection of Capsaicin into the tongue or in the lower gingiva stimulates pERK+ neurons localized in the dorsal half of the Vc, whereas the injection of Capsaicin into the anterior hard palate, into the upper gingiva, or into the buccal mucosa stimulates pERK+ neurons in both dorsal and ventral Vc. In the same way, the injection of Capsaicin into oral sites stimulates a large number of pERK+ neurons in the contralateral Vc [97].

The most common causes of chronic pain are ulcerated lesions by infections (HSPV-1), autoimmune diseases, trauma, or cancer. Recently, it has been shown that some patients affected by COVID-19 presented painful oral lesions, such as blisters or ulcers [98,99]. The last is mainly characterized by acute pain that may evolve into chronic pain. The most common ulcerated lesions of the mucosa include aphthous stomatitis, mechanical irritation from prostheses, radiation therapy for the treatment of head and neck cancer, or chemotherapy. The latter provokes, as a side effect, “oral mucositis”, which usually shows atrophy, swelling, erythema, and ulceration [100] for a prolonged period of time (>70 days). Mucosal ulceration causes spontaneous pain and mechanical hyperalgesia, through the release of prostaglandins and the activation of protease-activated receptor 2, leading to sensitization of TRPV1, TRPA1, and TRPV4 [101,102]. Therefore, the pharmacological inhibition of TRPV1 relieves hypersensitivity to heat. On the contrary, the inhibition of TRPA1 relieves cold hypersensitivity [93].

Trigeminal neuralgia (TN) is characterized by recurrent paroxysmal attacks similar to electric shocks in the area of trigeminal nerve innervation [103], often involving both facial skin and intraoral mucosa. TN can be triggered by physiologically harmless stimuli, such as tooth brushing, mouth opening, phonation, or chewing, or following the intake of acidic or spicy solutions [104].

Painful post-traumatic trigeminal neuropathy (PTTN) occurs after craniofacial or oral trauma. Iatrogenic causes include damage to the lingual nerve or to the lower alveolar nerve during the extraction of the third molar or the injection of a local anesthetic. PTTN induces moderate to severe pain or a burning sensation, which can be continuous. The most common manifestation of this syndrome is uncontrollable oral pain, generally localized both to the homolateral alveolar process and the homolateral dental hemiarch. In some cases, patients refer to a constant pain interrupted by unbearable poussées, often nocturnal. In other cases, PTTN occurs only with poussées without constant pain or with constant pain without poussées. Other clinical manifestations of PTTN are paresthesia, hypoesthesia, and anesthesia, localized to the area of second or third trigeminal branch innervation. The recovery time is unpredictable. Sometimes, functional alterations are irreversible. An anticonvulsant, such as carbamazepine, is recommended as a front-line treatment for TN, while a tricyclic antidepressant, such as amitriptyline, is used for PTTN.

It is interesting to note the substantial contribution of peripheral mechanisms in the treatment of oral pain; in fact, this painful symptomatology is resolved immediately in 67% of cases after the application of a local anesthetic spray, as well as after the extraoral or intraoral injection of botulinum toxin type A [105,106]. Likewise, topical administration of Capsaicin on the oral mucosa seems to improve chronic oral neuropathic pain, acting primarily on peripheral mechanisms [107]. This hypothesis is supported by a series of preclinical studies, in which Capsaicin represented an alternative treatment for chronic neuropathic pain, when the first-line treatment, based on an anticonvulsant or antidepressant, was not effective or could not be used due to side effects.

As a consequence of a lesion of the trigeminal nerve, large diameter trigeminal afferents become more sensitive to Capsaicin, suggesting increased expression of TRPV1 in non-nociceptive neurons [108].

Atypical odontalgia (AO) (PTTN, persistent idiopathic dentoalveolar pain or phantom tooth pain) is a condition of chronic pain after an endodontic treatment or tooth extraction in the dentoalveolar site, without clinical or radiographic signs [109]. AO behaves similar to peripheral neuropathy and its most frequent symptom is an increase in mechanical and cold painful perception [109]. This hyperalgesia can be attributed to the central mechanisms, as the administration of Capsaicin provokes greater pain in AO patients [1].

Another common condition is BMS, and it is characterized by other chronic painful conditions, such as headache, temporomandibular disorder (TMD), back pain, and fibromyalgia [110]. In patients with BMS, TRPV1 expression is increased in the remaining nerve fibers, and nerve growth factor (NGF) expression is increased in the basal nervous fibers of the epithelial layer. This may explain tongue hypersensitivity after the application of Capsaicin in BMS patients [111]. The efficacy of topical treatment based on clonazepam or Capsaicin in BMS patients suggests that the nociceptive afferents contribute to the reduction in pain induction or that they provoke nociceptive afferents’ degeneration [112,113].

In several clinical studies, the use of Capsaicin has been evaluated in oral rinse formulations with concentrations ranging from 0.025% to 0.075% [15]. The treatment showed good efficacy, improving BMS symptoms after only 7-day treatment with Capsaicin rinsing. The same result was not obtained through a placebo treatment [3].

Similarly, Petruzzi et al. [9] demonstrated a marked improvement in BMS using Capsaicin, even if the administration was systemic and occurred over a 4-week period. The adverse effects were recorded, with the most common being gastric pain, which limited the administration of this natural compound through this route.

In general, we can deduce that, despite the evident positive effects obtained from the use of Capsaicin in BMS management, there are limitations. In fact, even in topical use, a limited effect over time was recorded in terms of improvement [12]. Another limiting factor was the burning sensation produced by the use of the Capsaicin rinse, although the discomfort threshold varied among patients [13,14,114].

Painful symptomatology is increased in patients with resection of the chorda tympani nerve because they present upregulated expression of TRPV1 and TRPA1 in the geniculate ganglia [115,116]. This evidence demonstrated that there are interactions between the gustatory and nociceptive circuits since dysfunctional gustatory afferents can modulate oral pain. Furthermore, after applications of natural compounds such as Capsaicin and menthol, the oral mucosa can stimulate not only the trigeminal nerves but also the gustatory nerves. In humans, Capsaicin and menthol induce a bitter taste [116]. In fact, approximately 10–20% of the neurons of the geniculate ganglia express TRPV1 and TRPA1, and some neurons also express TRPM8 [117,118]. Therefore, the responses to intraoral Capsaicin can be interpreted as neuroplasticity, affecting both the gustatory and somatosensory pathways. In addition, the therapeutic effects of Capsaicin for pain treatment in BMS patients can be attributed to the desensitization of the gustatory nerves that express TRPV1.

The TRPV1 channel is also activated by the release of neuropeptides such as CGRP, released at the level of the dental pulp and periradicular tissues, innervated by Capsaicin-sensitive neurons. In animal studies, a protective role was discovered for the initial phase of apical periodontitis after an injection of Capsaicin or even a single subcutaneous administration for the specific deletion of peptidergic neurons [119].

Recently, a sensory role of TRPV1 channels was discovered in non-neuronal cells, such as keratinocytes [120]. In particular, TRPV1 channels’ epithelia activation affects numerous biological processes, including proliferation, differentiation, and apoptosis [121,122].

Gingival epithelial cells (GECs) make a great contribution to the homeostasis of periodontal tissues, as they form a physical barrier. A break in this epithelial barrier involves the invasion of exogenous substances into the gum, favoring the onset of periodontal diseases. Through in vitro and in vivo studies, it was discovered that TRPV1 signaling in GECs, following Capsaicin treatment, may induce the transcriptional upregulation of growth factors and may concur to periodontal tissue homeostasis by triggering a positive feedback loop, involving increased proliferation [123].

Recently, TRPV1 expression was reported in secretory epithelia. Capsaicin seems to improve salivary secretion both in humans and in a rabbit model [124,125,126,127] by directly affecting the paracellular permeability of the tight junctions (TJs) [128]. Capsaicin application reduced TJs’ expression and their destruction in transplanted submandibular glands [129,130].

Capsaicin can also indirectly increase salivary secretion. Topically, its application around the mouth skin increased salivary secretion from the submandibular and sublingual glands in humans [126]. In a study conducted on patients with hyposalivation, Capsaicin intensified the salivary secretion in healthy volunteers compared to subjects with dry mouth related to hyposalivation [127].

The muscarinic acetylcholine receptors (mAChRs) are an upstream mediator of salivary secretion [131]. Their distribution is as follows: M1 and M3 receptors are expressed in the sublingual and submandibular glands; in addition, M3 receptor expression is predominant in the parotid gland [132]. The activation of mAChRs mediates the [Ca^2+^]_i_ mobilization from endoplasmic reticulum storage. The increased [Ca^2+^]_i_ level induces saliva secretion by activating calcium-activated chloride channels (CACC) [133,134]. In this mechanism, the TRPV1 channel is independently involved. In particular, the chemical or physical stimulation causes the increase in the [Ca^2+^]_i_ level by TRP channels [135].

In an in vitro study, it has been observed that Capsaicin enhances [Ca^2+^]_i_ levels in cells isolated from the human submandibular gland (SMG) [124]. On the contrary, in rabbit models, capsazepine blocked salivation after Capsaicin treatment in isolated SMGs, confirming that TRPV1 mediates Capsaicin-induced saliva secretion [124].

In addition to the role of TRPV1 in saliva secretion, the interaction between TRPV and CACC has also been explored. The chemical sensation or taste are strong agonists of salivation. It has been found that Capsaicin inhibits outward-rectifying K^+^ channels in taste receptor cells (TRCs), isolated from circumvallate papillae in rats. The inhibition of the K^+^ channel may induce TRC excitation, activating the solitary nucleus tract (SNT) in the brain [136]. Various studies have reported that Capsaicin dynamically modulates the permeability of both TRPV1 routes [137].

Another relevant topic is oral carcinogenesis, which is induced by many factors, among which is diet. Oral squamous cell carcinoma (OSCC) is one of the most widespread cancers in the world, whose incidence is highly variable. OSCC records low rates in some countries, such as Mexico, where people consume a large number of chilies daily [138,139,140]. In fact, although initial investigations suggested that a continuous intake of Capsaicin increased the risk of developing carcinomas of the digestive system, it was later discovered that Capsaicin could have a chemo-preventive action by altering the microsomal function of several enzymes, which are essential for the metabolic activation and elimination of multiple mutagens [141,142].

Capsaicin seems to act as a cancer-suppressing agent through its antioxidant and anti-inflammatory action, blocking several signaling pathways [32].

In addition, a lower incidence of oral epithelial dysplasia (OED) is reported after Capsaicin treatment [6]. The presence of OED represents the key prognostic factor for predicting the malignant transformation of an oral mucosal lesion [6,30].

Considering all available information, we can speculate that dietary Capsaicin consumption could provide a chemo-preventive effect for OSCC [143].

### Our Experience with Capsaicin in Dentistry

It is interesting to propose our personal experience, which has matured during the last few years, about Capsaicin topical application in PTTN. In this manuscript, we present five clinical cases, in which topical application of Capsaicin gel was used to treat uncontrollable oro-facial pain of iatrogenic origin.

All patients developed PTTN after post-endodontic treatment and all of them were previously treated with infraorbital nerve or sphenopalatine ganglion block, using 1 mL of 0.5% lidocaine or 1 mL of 0.5% mepivacaine with a 27-gauge needle, but only with transitory benefits. Patients were then treated with repeated courses of radio frequency (RF) pulses with a 24-gauge needle on their terminal nerve, also without substantial benefits. After these procedures, patients were treated with a topical application of Capsaicin gel.

Before the beginning of Capsaicin treatment, we performed, as common clinical practice, an oral examination to evaluate the presence of:

- Mucosal hyperemia.
- Acupressure with fovea sign or transient ischemia of the oral mucosa.
- Swollen or raised mucosa.
- Tactile allodynia.
- Thermic allodynia performed with a wet cotton wad at a known temperature (42 °C for hot and 2–4 °C for cold).

We tested patients’ sensibility to vanilloid receptors using a stress test with methyl acetate. All patients were sensitive to menthol and therefore to Capsaicin.

Capsaicin gel was applied to the painful mucosal area, three times a day, every 8 h. We used increasing concentrations of Capsaicin, starting from 0.01%, through to 0.05% and up to 0.5%. Patients indicated a total remission of pain within 5 min after the application and mild hypersensitivity after 120 min. After 4 h, the pain reappeared, and another application was necessary.

No patients presented adverse local or systemic effects. Capsaicin seemed to be well-tolerated by patients, despite local burning pain and other local side effects observed during the first applications.

Currently, there is no contraindication in the use of local Capsaicin, except the presence of mucosal lesions at the site of application. No cases of intoxication or systemic side effects related to local Capsaicin use are known.

Even if more clinical studies are needed to confirm these data, local Capsaicin application is sufficiently safe and effective for the treatment of oral neurosensitive pain, and it represents a promising method for the management of neuropathic oral diseases.

## 7. Conclusions

Since ancient times, Capsaicin has been used for its particular beneficial properties. However, only recently have its mechanisms of action and potentialities been highlighted. In addition to the healthy properties already studied (namely, the improvement of dyslipidemia, the beneficial effects in chronic kidney disease, rather than the improvement of insulin sensitivity or the reduction in body weight in obese subjects, etc.), Capsaicin would seem to be a valid therapeutic option for the treatment of PTTN.

In this regard, we present our experience in the local use of Capsaicin in the treatment of PTTN. Initially, in our study population, Capsaicin’s contact with oral mucosa caused hyperalgesia and increased neuropathic pain in some patients. However, its continuative application desensitized neurosensitive fibers, showing pain relief.

In conclusion, Capsaicin seems to be well-tolerated by patients, despite the local burning pain and other minor local side effects observed during the first applications, making it a valid new therapeutic agent, free from side effects, not only in internal medicine but also in dentistry.

## List of Abbreviations

ACEAngiotensin-converting-enzymeAHArterial hypertension AOAtypical odontalgiaBMSBurning mouth syndromeCACCCalcium-activated chloride channelsCGRPCalcitonin gene-related peptideCKDChronic kidney diseaseCVDCardiovascular diseaseEGFEpidermal growth factorFDAFood and drug administrationGFRGlomerular filtration rateGECsGingival epithelial cells GLUT2Glucose transporter 2HDLHigh-density lipoproteinsHIV-DSPNHIV-associated distal sensory polyneuropathy HOMA-IRHomeostasis model assessment of insulin resistanceJAK/STATJanus kinase/signal transducer and activator of transcription LDLLow-density lipoproteinsLXRLiver X receptormAChRsMuscarinic acetylcholine receptors NF-kBNuclear factor-kB NGFNerve growth factorNONitric oxideOH-1Heme oxygenase-1OEDOral epithelial dysplasia PDX-1Pancreatic duodenal homebox-1pERKphosphorylated kinasePHNPost-herpetic neuropathyPI3/AKTPhosphoinositide 3-kinase/protein kinase BPPARαPeroxisome proliferators-activated receptor-αPTNNPainful post-traumatic trigeminal neuropathyomaSMGSubmandibular glandSNTSolitary nucleus tractSPSubstance PTAK1/MAPKTransforming growth factor-β-activated kinase 1/mitogen-activated protein-kinase TGTriglyceridesTJsTight junctionsTM1-66 transmembrane regions TMDTemporomandibular disorderTNTrigeminal neuralgiaTNF-αTumor necrosis factor-α TRCsTaste receptor cellsTRPVTransient receptor potential vanilloidVcCaudal trigeminal subnucleus

## Data Availability

Not applicable.
